# The PB2 Subunit of the Influenza A Virus RNA Polymerase Is Imported into the Mitochondrial Matrix

**DOI:** 10.1128/JVI.01384-16

**Published:** 2016-09-12

**Authors:** Joshua C. D. Long, Ervin Fodor

**Affiliations:** Sir William Dunn School of Pathology, University of Oxford, Oxford, United Kingdom; St. Jude Children's Research Hospital

## Abstract

The polymerase basic 2 (PB2) subunit of the RNA polymerase complex of seasonal human influenza A viruses has been shown to localize to the mitochondria. Various roles, including the regulation of apoptosis and innate immune responses to viral infection, have been proposed for mitochondrial PB2. In particular, PB2 has been shown to inhibit interferon expression by associating with the mitochondrial antiviral signaling (MAVS) protein, which acts downstream of RIG-I and MDA-5 in the interferon induction pathway. However, in spite of a growing body of literature on the potential roles of mitochondrial PB2, the exact location of PB2 in mitochondria has not been determined. Here, we used enhanced ascorbate peroxidase (APEX)-tagged PB2 proteins and electron microscopy to study the localization of PB2 in mitochondria. We found that PB2 is imported into mitochondria, where it localizes to the mitochondrial matrix. We also demonstrated that MAVS is not required for the import of PB2 into mitochondria by showing that PB2 associates with mitochondria in MAVS knockout mouse embryo fibroblasts. Instead, we found that amino acid residue 9 in the N-terminal mitochondrial targeting sequence is a determinant of the mitochondrial import of PB2, differentiating the localization of PB2 of human from that of avian influenza A virus strains. We also showed that a virus encoding nonmitochondrial PB2 is attenuated in mouse embryonic fibroblasts (MEFs) compared with an isogenic virus encoding mitochondrial PB2, in a MAVS-independent manner, suggesting a role for PB2 within the mitochondrial matrix. This work extends our understanding of the interplay between influenza virus and mitochondria.

**IMPORTANCE** The PB2 subunit of the influenza virus RNA polymerase is a major determinant of viral pathogenicity. However, the molecular mechanisms of how PB2 determines pathogenicity remain poorly understood. PB2 associates with mitochondria and inhibits the function of the mitochondrial antiviral signaling protein MAVS, implicating PB2 in the regulation of innate immune responses. We found that PB2 is imported into the mitochondrial matrix and showed that amino acid residue 9 is a determinant of mitochondrial import. The presence of asparagine or threonine in over 99% of all human seasonal influenza virus pre-2009 H1N1, H2N2, and H3N2 strains is compatible with mitochondrial import, whereas the presence of an aspartic acid in over 95% of all avian influenza viruses is not, resulting in a clear distinction between human-adapted and avian influenza viruses. These findings provide insights into the interplay between influenza virus and mitochondria and suggest mechanisms by which PB2 could affect pathogenicity.

## INTRODUCTION

Viruses target mitochondrial processes during infection to promote their own replication (1, 2). Given that mitochondria act as central hubs for energy production and distribution, apoptosis, and innate immune responses, this is perhaps not surprising. Mitochondria are best known for their role in oxidative ATP synthesis, which requires the generation of a mitochondrial membrane potential (MMP) across the mitochondrial inner membrane. However, they also contain cell death mediators and are involved in the induction of cytokine expression (3, 4).

One example of a process which is widely modulated by viruses is the induction of apoptotic cell death. By inhibiting cell death, viruses can increase their ability to replicate or induce latency. On the other hand, viruses can increase virion release and dissemination by promoting cell death (1, 2). The induction of apoptosis is normally triggered by permeabilization of the mitochondrial membranes; a process controlled by the Bcl-2 family of proteins. Viruses such as hepatitis B virus and human adenovirus encode apoptosis-regulating proteins with Bcl-2 homology which can be either pro- or antiapoptotic in nature (5, 6). Another targeted process is the induction of interferon (IFN) expression in response to viral infection. Interferon triggers an antiviral state in cells and thus limits the replication and spread of viruses (7). The interferon induction pathway signals via the mitochondrial antiviral signaling protein (MAVS), which is found on the mitochondrial outer membrane (8–11). Multiple viruses act to dampen the signaling in this pathway. One example is hepatitis C virus, which expresses a protein that causes cleavage of MAVS and thus limits interferon release (11). Beyond this, mitochondrial processes such as calcium homeostasis, the maintenance of the MMP, and the generation of reactive oxygen species have also been found to be affected by viruses (2).

Polymerase basic 2 (PB2) protein is a component of the influenza A virus RNA-dependent RNA polymerase complex alongside the polymerase basic 1 (PB1) and polymerase acidic (PA) protein subunits (12, 13). The viral polymerase carries out transcription of viral genes and replication of the viral RNA genome in the nucleus of infected cells (14). However, PB2 of some influenza virus strains has also been found to localize to the mitochondria, independently of PB1 and PA (15, 16). An N-terminal mitochondrial targeting sequence (MTS) was found to be responsible for the mitochondrial localization of PB2 (16, 17). Despite high sequence conservation in this region, position 9 shows variations in influenza A viruses of different hosts and is a determinant of the mitochondrial localization of PB2 (15, 18). Most human seasonal influenza virus pre-2009 H1N1, H2N2, and H3N2 strains encode mitochondrial PB2 with asparagine at position 9 (N9-PB2) (15). On the other hand, the majority of avian influenza viruses encode nonmitochondrial PB2 with an aspartic acid in this position (D9-PB2). Interestingly, the swine origin pandemic H1N1 influenza viruses that appeared in the human population in 2009 encode nonmitochondrial D9-PB2, in agreement with the avian origin of the PB2 segment of these viruses (19).

The exact location of PB2 at the mitochondria is unknown, and the functional significance of the mitochondrial localization of PB2 remains obscure. Recombinant influenza A/WSN/33 viruses expressing PB2 with mutations at L7 and L10 in the MTS, rendering PB2 nonmitochondrial, showed reduced growth in cell culture and animal models and induced more MMP loss, suggesting that mitochondrial PB2 may contribute to the preservation of mitochondrial function during influenza virus infection (16). Further studies of recombinant influenza A/WSN/33 viruses encoding wild-type mitochondrial N9-PB2 or mutant nonmitochondrial D9-PB2 implicated PB2 in the regulation of host antiviral innate immune pathways. In particular, the mutant virus encoding nonmitochondrial PB2 was found to induce higher levels of beta interferon (IFN-β) in cell culture and was attenuated in a mouse infection model (15). In agreement with this, a D9N mutation in the PB2 of an avian H5N1 virus, which drives PB2 to the mitochondria, resulted in increased virulence in mice (20). It has been proposed that PB2 is involved in regulating innate immune pathways by interacting with MAVS (15, 21). During infection with influenza virus, viral RNA species are detected by the cellular DEAD/box helicase RIG-I (22). This leads to the activation of RIG-I, resulting in its interaction with MAVS at the mitochondrial outer membrane. Activated MAVS forms signaling-competent oligomers on the mitochondrial surface, which interact with downstream adapters and kinases, leading to the induction of interferon expression (23). The N-terminal 242-amino-acid (aa) region of PB2 is thought to be mainly responsible for the binding to MAVS (21, 24). Both the mitochondrial and nonmitochondrial forms of PB2 have been shown to associate with MAVS, and both forms inhibit IFN-β expression when overexpressed in cells (15, 25).

In this study, we aimed to further investigate the mitochondrial association of PB2. We show that the mitochondrial localization of PB2 is not mediated by the association of PB2 with the previously reported PB2 interactor MAVS at the outer mitochondrial membrane but by an MTS at the N terminus of PB2. The MTS drives the import of PB2 into the mitochondria and specifically into the mitochondrial matrix space. We show that mitochondrial PB2 affects the mitochondrial fusion/fission equilibrium, leading to the fragmentation of mitochondria, and that a recombinant influenza A virus encoding nonmitochondrial PB2 is attenuated in cell culture in a MAVS-independent manner. These findings highlight the potential of a matrix-associated function of PB2 during influenza virus infections.

## MATERIALS AND METHODS

### Plasmids.

Plasmids pcDNA-PB2 and pcDNA-PB2-GFP (pcDNA-PB2-green fluorescent protein) expressing wild-type or N9D mutant PB2 of the influenza A/WSN/33 virus (H1N1) have been described previously (15, 26, 27). The plasmid expressing N9T mutant PB2 with a C-terminal GFP tag was generated via site-directed mutagenesis of the pcDNA-PB2-GFP plasmid. To generate plasmids expressing ascorbate peroxidase (APEX)-tagged PB2, the APEX sequence was PCR amplified from the MTS-APEX plasmid (a kind gift of Alice Y. Ting, Massachusetts Institute of Technology) (28) and ligated into the pcDNA-PB2-GFP plasmids, replacing the GFP open reading frame.

### Fluorescence microscopy.

Mouse embryonic fibroblasts (MEFs) (a kind gift of Caetano Reis a Sousa, Francis Crick Institute), generated from either wild-type or MAVS^−/−^ mice, were grown on glass coverslips in 6-well plates. Cells were transfected with 2.5 μg of plasmids expressing PB2 or PB2-GFP using Lipofectamine 2000 following the instructions of the manufacturer (Invitrogen). Cells were stained at 24 h posttransfection for 30 min with 10 nM Mitotracker Red (Molecular Probes) in cell culture medium, washed for 2 min with fresh medium, and fixed with 3.5% formaldehyde–medium. Coverslips were washed three times for 5 min in phosphate-buffered saline (PBS) and mounted in Mowiol. For immunofluorescence experiments, Vero cells grown on coverslips were transfected with plasmids expressing PB2, stained with 100 nM Mitotracker Red, and fixed as described above. Cells were then blocked with 2% fetal bovine serum (FBS)–PBS (blocking buffer) overnight at 4°C and then permeabilized for 7.5 min using 0.2% Triton X-100–PBS. Cells were stained with a rabbit polyclonal anti-PB2 antibody (16) and a Cy2-conjugated anti-rabbit secondary antibody (Jackson ImmunoResearch Laboratories). Coverslips were mounted in Mowiol, and images were obtained with an Axioplan 2 microscope. Image analysis was carried out using ImageJ software.

### Electron microscopy.

HEK 293T cells, transfected in suspension with 1 μg of the relevant plasmid using Lipofectamine 2000, were grown on plastic coverslips (Thermonox) in 24-well plates. MTS-APEX-expressing cells incubated for 1 day, or PB2-APEX-expressing cells incubated for 2 days, were fixed in prewarmed fixative solution (2.5% formaldehyde, 2% paraformaldehyde, 0.1 M sodium cacodylate [pH 7.4], 2 mM CaCl_2_) for 1 h. Cells were washed five times with wash buffer (0.1 M sodium cacodylate [pH 7.4], 2 mM CaCl_2_) and once with wash buffer containing 1.5 mg/ml glycine, followed by five washes in wash buffer at 4°C. Cells were incubated with wash buffer containing 0.5 mg/ml diaminobenzidine and 0.03% H_2_O_2_ for 4 h. Cells were washed five times with wash buffer, stained with 2% osmium tetroxide, washed three times in distilled water, and then stained with 0.5% uranyl acetate. The samples were dehydrated in a cold ethanol series of ethanol concentrations increasing to 100%. Resin infiltration was then completed with a graded resin series of increasing resin concentrations. The resin was left to polymerize at 60°C for 48 h. Sections were generated using a Leica UC7 microtome, mounted on 200-mesh copper grids, and poststained with Reynold's lead citrate. Sections were imaged using a Tecnai 12 transmission electron microscope (TEM) with a Gatan US100 charge-coupled-device (CCD) camera. Densitometry and mitochondrial size analyses were carried out using ImageJ. The transfection efficiency achieved in HEK 293T cells with Lipofectamine 2000 was about 50% to 60%.

## RESULTS

### Mitochondrial localization of PB2 is not mediated by its binding to MAVS.

**FIG 1:**
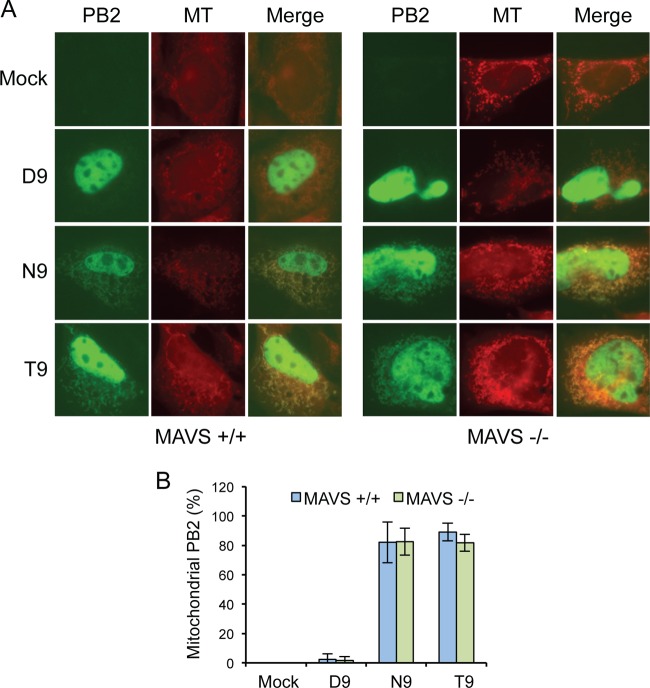
MAVS is not required for the mitochondrial localization of PB2. (A) Cellular distribution of GFP-tagged PB2 with D9, N9, or T9 in transfected MAVS^+/+^ and MAVS^−/−^ MEF cells. Cells were stained with the fluorescent mitochondrial marker Mitotracker (MT) and imaged by fluorescence microscopy. (B) PB2-expressing cells were scored for mitochondrial localization of PB2. Column data represent the percentages of PB2-expressing cells with mitochondrial PB2 signal. Bars represent standard deviations of averages of data from three independent experiments (*n* = 17 to 21 cells/experiment).

Previous work demonstrated that PB2 interacts with the outer mitochondrial membrane protein MAVS (15, 21, 24), and recent work provided experimental data showing that knockdown of MAVS by small interfering RNA (siRNA) decreases the association of PB2 with mitochondria (29). Together, these results suggest that the mitochondrial association of PB2 might be mediated via its interaction with MAVS at the outer mitochondrial membrane. In other studies, however, both mitochondrial PB2 and nonmitochondrial PB2 were found to associate with MAVS, suggesting that mitochondrial targeting of PB2 is independent of its interaction with MAVS (15, 25). To test whether association with MAVS is required for the mitochondrial targeting of PB2, we expressed GFP-tagged nonmitochondrial D9-PB2 or mitochondrial N9-PB2 in wild-type MEFs and MAVS^−/−^ MEFs and determined their localization by fluorescence microscopy. We also included T9-PB2 in the analysis, as about 9% of seasonal pre-2009 H1N1, H2N2, and H3N2 human influenza viruses encode T9 rather than the more typical N9. The cells were stained with Mitotracker to determine the colocalization of PB2 with the mitochondria. As expected, in wild-type MEFs, all three PB2s showed strong nuclear localization (Fig. 1A), as PB2 contains a nuclear localization sequence (30). As previously reported (15), there was no detectable GFP signal outside the nucleus of cells expressing D9-PB2. However, both N9-PB2 and T9-PB2 showed mitochondrial localization in the vast majority of cells (Fig. 1). This means that over 99% of all human seasonal influenza virus pre-2009 H1N1, H2N2, and H3N2 strains encode a mitochondrion-localizing PB2. This is in contrast to avian influenza viruses, the vast majority of which encode a nonmitochondrial D9-PB2. Both N9-PB2 and T9-PB2 showed the same mitochondrial localization pattern in MAVS^−/−^ MEFs as in the corresponding wild-type MEFs (Fig. 1), indicating that the interaction between PB2 and MAVS is not required for the localization of PB2 to mitochondria.

### Position 9 controls the import of PB2 into the mitochondria.

**FIG 2:**
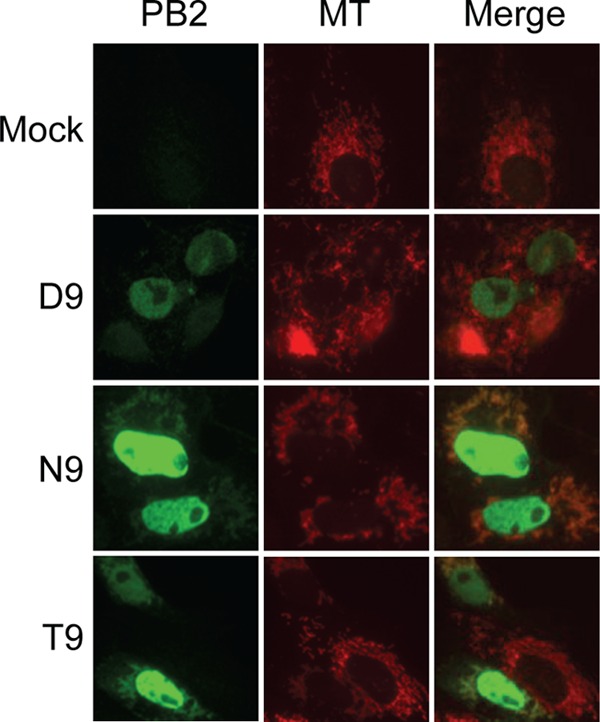
The APEX tag has no effect on the cellular distribution of PB2 with D9, N9, or T9 in Vero cells. Cells were stained with the fluorescent mitochondrial marker Mitotracker (MT), and APEX-tagged PB2 was detected using an anti-PB2 antibody. Cells were imaged by fluorescence microscopy.

We therefore investigated what other mechanisms could be mediating PB2 localization to the mitochondria and where exactly this mitochondrial population of PB2 was localized. We hypothesized that aspartic acid at position 9 of PB2 was affecting mitochondrial localization by disrupting an N-terminal MTS which would otherwise mediate import of PB2 into the mitochondria. Position 9 of PB2 is found within an N-terminal amphipathic helix with a number of leucines, serines, and arginines. N-terminal amphipathic helices can act as MTSs, and proteins with amphipathic helices have also been found to be inserted into the phospholipid monolayer of lipid droplets or lipid membranes (31–36). In order to determine the submitochondrial localization of PB2, we tagged PB2 with the enhanced ascorbate peroxidase (APEX) tag (28). Upon addition of H_2_O_2_, APEX catalyzes the oxidative polymerization of diaminobenzidine (DAB) to generate a cross-linked and locally deposited membrane-impermeant precipitate which can be detected directly by light microscopy or, after staining, by electron microscopy. Fusion of APEX to a protein of interest allows the suborganelle localization of the protein to be determined. Imaging of cells expressing the PB2-APEX fusion proteins with D9, N9, or T9 by immunofluorescence against PB2 confirmed that the APEX tag did not interfere with their intracellular localization. Thus, all three fusion proteins predominantly localized to the nucleus, with N9-PB2 and T9-PB2 also localizing to the mitochondria, while no mitochondrial localization was observed for D9-PB2 (Fig. 2).

**FIG 3:**
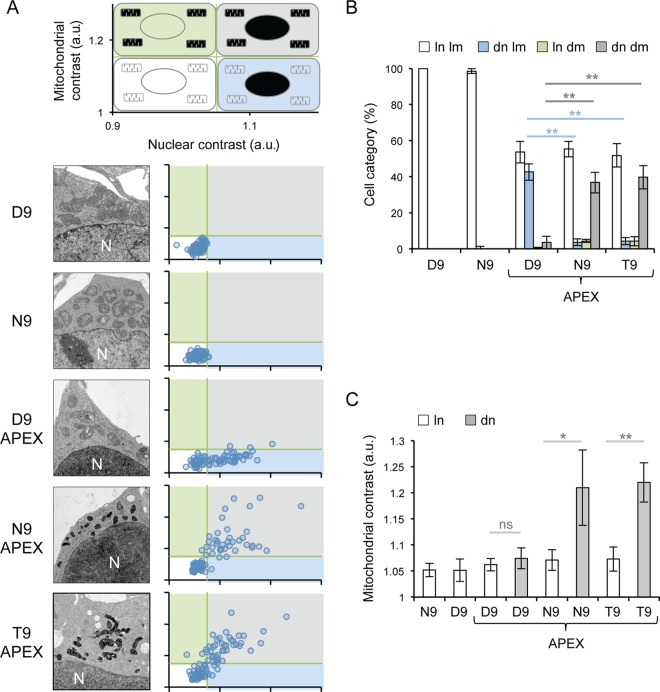
PB2s with N9 or T9 are imported into mitochondria. (A) HEK 293T cells expressing untagged PB2 with D9 or N9 or APEX-tagged PB2 with D9, N9, or T9 were analyzed by electron microscopy. Scatter plots show the results of densitometry analysis of the mitochondrial and nuclear contrast of individual cells relative to their cytoplasmic contrast. Each point represents a single cell. Data were gated into categories of low and high mitochondrial contrast (*y* axis) and low and high nuclear contrast (*x* axis) based on the contrast of mitochondria and nuclei of cells expressing untagged PB2. A diagrammatic key displaying axis titles and values for scatter plots is shown at the top. Staining phenotypes of cells for each quadrant are also shown (white, light mitochondria/light nucleus; blue, light mitochondria/dark nucleus; green, dark mitochondria/light nucleus; gray, dark mitochondria/dark nucleus). N, position of the nucleus; a.u., arbitrary units. (B) Quantitative analysis of cells expressing untagged PB2 with D9 or N9 or APEX-tagged PB2 with D9, N9, or T9, categorized as having either light nuclei (ln) or dark nuclei (dn) and light mitochondria (lm) or dark mitochondria (dm), based on the gating performed as described for panel A. Column data represent percentages of cells expressing the indicated PB2 belonging to the indicated staining categories. Bars represent standard deviations of averages of data from three independent experiments. Asterisks indicate a significant difference between samples (one-sample *t* test; **, *P* < 0.01). (C) Comparison of mitochondrial signals of cells with light (ln) or dark (dn) nuclei expressing the indicated PB2, based on the gating performed as described for panel A. Column data represent averages of mitochondrial contrast data from each population. Bars represent the standard deviations of averages of contrast data from three independent experiments. Asterisks indicate a significant difference between samples (one-sample *t* test; **, *P* < 0.01; *, *P* < 0.05; ns, *P* > 0.05).

For analysis by electron microscopy, cells expressing PB2-APEX fusion proteins were treated with H_2_O_2_ and DAB and stained with osmium tetroxide and uranyl acetate. All cells expressing PB2-APEX displayed strong nuclear staining (Fig. 3A). However, cells expressing N9-PB2-APEX and T9-PB2-APEX also displayed strong staining in the mitochondria. No strong contrast was observed in cells expressing untagged D9- or N9-PB2s. For quantitative evaluation, imaged cells were analyzed by calculating the contrast of each cell's nucleus and mitochondria relative to that of its cytoplasm. The data point from each cell was plotted onto a scatter plot of increasing nuclear contrast (*x* axis) against increasing mitochondrial contrast (*y* axis) (Fig. 3A). Cells expressing untagged D9-PB2 and N9-PB2 exhibited low mitochondrial and nuclear contrast. When cells were transfected with the D9-PB2-APEX construct, a population of cells with low mitochondrial and low nuclear contrast, likely representing untransfected cells, was observed. However, we observed a second population of cells which had increased nuclear staining with no associated increase in mitochondrial contrast, demonstrating the localization of PB2 solely to the nucleus. A similar population of cells with increased nuclear staining was observed in N9-PB2-APEX- and T9-PB2-APEX-expressing cell populations; however, these cells also showed an increase in mitochondrial contrast. In order to combine the data from three independent experiments, two gates were produced to sort cells into four categories based on mitochondrial contrast (low or high) and nuclear contrast (low or high). These gates were placed so that the data points for cells expressing untagged N9-PB2 and D9-PB2 were found in the low-contrast/low-contrast quadrant. A schematic key explaining the gating strategy and which type of cell is found under each set of conditions is shown (Fig. 3A, top). This quantitative evaluation of the data revealed that D9-PB2-APEX expression led to increased nuclear staining whereas T9-PB2-APEX and N9-PB2-APEX expression caused increased nuclear and mitochondrial staining (Fig. 3B). Together, the data demonstrate that N9-PB2 and T9-PB2, as well as containing a nuclear localization signal, also contain an MTS which drives their import into mitochondria.

Finally, to quantitatively evaluate whether or not there was any import of D9-PB2-APEX into mitochondria, cells from the three independent experiments performed for each construct were separated into groups of cells with light or dark nuclei using the gating parameters shown in Fig. 3A and then the average mitochondrial contrast for each group was calculated. As expected, the cells with dark nuclei expressing N9-PB2-APEX or T9-PB2-APEX had a higher average level of mitochondrial contrast than the cells with light nuclei (Fig. 3C). However, there was no significant increase in mitochondrial contrast in cells with dark nuclei expressing D9-PB2-APEX compared to cells with light nuclei. Therefore, there was no detectable import of D9-PB2 into the mitochondria. Taken together, these data show that the vast majority of PB2s encoded by human seasonal influenza virus pre-2009 H1N1, H2N2, and H3N2 strains is imported into the mitochondria and that position 9 of PB2 is a determinant of the mitochondrial import.

### PB2 is imported into the mitochondrial matrix.

**FIG 4:**
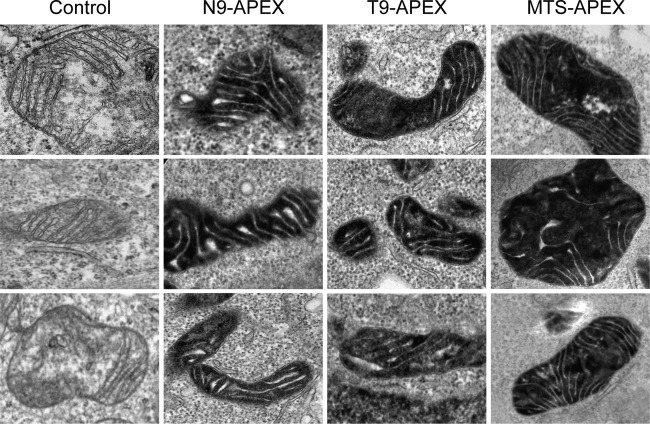
PB2 is imported into the mitochondrial matrix. HEK 293T cells expressing APEX-tagged PB2 with N9 or T9 or MTS-APEX data were analyzed by electron microscopy. Three representative images of mitochondria are shown for each construct.

In order to determine the submitochondrial localization of PB2, high-contrast mitochondria of cells expressing N9-PB2-APEX or T9-PB2-APEX were imaged at high magnification. Mitochondria are formed by a pair of membrane bilayers which separate two distinct regions. The first of these, termed the matrix space, is the area surrounded by the mitochondrial inner membrane and contains the mitochondrial DNA and mitochondrial ribosomes. The inner membrane, which is highly folded, contains the proteins of the electron transport chain. The inner membrane is surrounded by the outer mitochondrial membrane, resulting in a space between the two membranes termed the intermembrane space. The intermembrane space includes the volume formed by the invaginations in the inner membrane called “cristae” (1, 37). High-contrast mitochondria from N9-PB2-APEX- and T9-PB2-APEX-expressing cells were found to show high contrast in the mitochondrial matrix but not in the intermembrane space, as indicated by the presence of low-contrast invaginations of cristae (Fig. 4). A similar staining pattern was observed for the MTS-APEX construct, which is known to be targeted to the mitochondrial matrix space (28). Together, these data show that PB2 is found in the mitochondrial matrix space.

### Mitochondrial PB2 causes changes in mitochondrial morphology.

**FIG 5:**
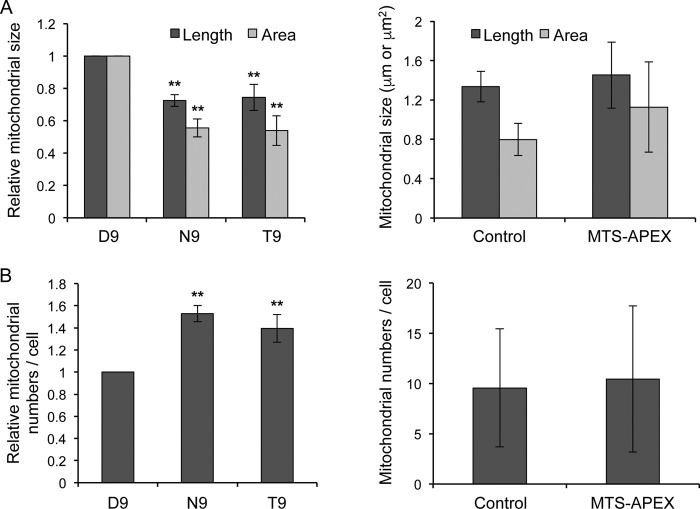
Mitochondria of cells expressing mitochondrial PB2 show reduced size and increased numbers compared to cells expressing nonmitochondrial PB2. Mitochondria from cells expressing APEX-tagged PB2 with D9, N9, or T9 or MTS-APEX or from untransfected cells (Control) were analyzed by measuring their greatest diameter and cross-sectional area (A) and by counting their numbers per cell (B). For APEX-tagged PB2 with D9, N9, or T9, 75 to 200 mitochondria from 13 to 15 cells per condition were analyzed (left panels). Column data represent relative mitochondrial length or area data (A) or mitochondrial numbers per cell (B) from three independent experiments, with data normalized against D9-PB2-APEX. Bars represent standard deviations. Asterisks indicate a significant difference between the marked sample and the corresponding D9 sample (one-sample *t* test; **, *P* < 0.01). For MTS-APEX and untransfected cells (Control), 94 and 67 mitochondria from 9 and 7 cells, respectively, were analyzed (right panels). Column data represent mitochondrial length or area (A) or mitochondrial numbers per cell (B). Bars represent standard deviations.

Mitochondria of cells expressing D9-PB2-APEX, N9-PB2-APEX, T9-PB2-APEX, or MTS-APEX were analyzed for morphological changes by measuring their maximum length and area, as well as by counting their numbers. The analysis revealed a significant reduction in the average size of the high-contrast mitochondria found in N9-PB2-APEX- or T9-PB2-APEX-expressing cells relative to that of the low-contrast mitochondria found in D9-PB2-APEX-expressing cells (Fig. 5A, left). This decrease in mitochondrial size was accompanied by an increase in average mitochondrial numbers per cell (Fig. 5B, left). These differences, however, were not observed in cells expressing MTS-APEX relative to untransfected cells, indicating that they were not caused by the import of the APEX tag into mitochondria (Fig. 5A and B, right). These observations suggest that PB2 imported into the mitochondrial matrix disrupts the mitochondrial fusion/fission equilibrium, leading to mitochondrial fragmentation, possibly through affecting the MMP, which has been found to be important for the maintenance of the fusion/fission equilibrium (16, 38).

### A recombinant influenza A virus encoding a nonmitochondrial PB2 replicates to lower titers in cell culture in a MAVS-independent manner.

**FIG 6:**
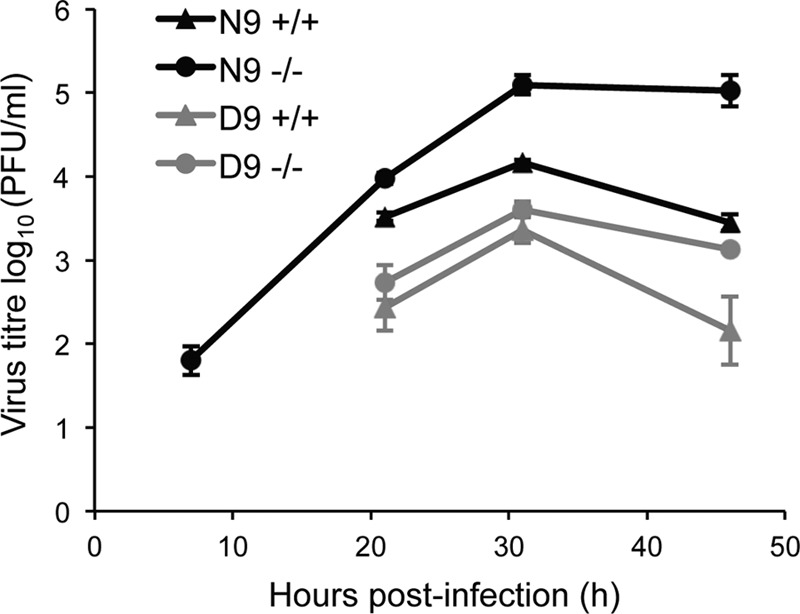
Influenza A virus encoding a mutant nonmitochondrial PB2 replicates to lower titers in MEFs in a MAVS-independent manner. Wild-type MEFs (+/+) or MAVS knockout MEFs (−/−) were infected with either wild-type A/WSN/33 virus expressing mitochondrial PB2 (N9) or a mutant isogenic virus expressing nonmitochondrial PB2 (D9) at a multiplicity of infection (MOI) of 0.01 and incubated at 37°C. Aliquots of cell supernatant were collected at 7, 21, 32, and 46 h postinfection, followed by a plaque assay in Madin-Darby bovine kidney (MDBK) cells. Data points represent the average viral titers from three infections; bars represent standard deviations.

To investigate the importance of mitochondrial PB2 for virus growth in cell culture, we infected wild-type MEFs with recombinant A/WSN/33 (H1N1) influenza A viruses which encode either a wild-type mitochondrial N9-PB2 (N9-WSN) or a mutant nonmitochondrial D9-PB2 (D9-WSN), and the growth kinetics of the two viruses were analyzed. Furthermore, in order to differentiate any effects caused by the interaction of PB2 with MAVS or the localization into the mitochondrial matrix, we also analyzed the growth kinetics of the two viruses in MAVS^−/−^ MEFs. The analysis revealed that the wild-type N9-WSN virus replicated to higher titers than the mutant D9-WSN virus in both wild-type and MAVS^−/−^ MEFs (Fig. 6). Interestingly, it was also found that the knockout of MAVS promoted the replication of N9-WSN but not that of D9-WSN (Fig. 6). These observations first suggest that the expression of mitochondrial matrix-localizing PB2 promotes the replication of influenza A viruses in mammalian cells. Second, given that N9-WSN also replicated to higher titers than D9-WSN in MAVS^−/−^ MEFs, the data suggest that the difference between N9-WSN and D9-WSN is not solely due to possible differential levels of induction of interferon by the two viruses. In summary, in mammalian cells, the expression of mitochondrial matrix localizing PB2 promotes the replication of influenza A virus in a manner distinct from the role of PB2 in suppressing the induction of IFN through its interaction with MAVS.

## DISCUSSION

In this paper, we report that the influenza virus PB2 polymerase subunit that colocalizes with mitochondria is imported into the mitochondrial matrix. Using electron microscopy and PB2 proteins fused to an APEX tag, which generates a membrane-impermeant product that can be stained with heavy metals, we found that mitochondrial PB2 is targeted to the mitochondrial matrix. We also found that amino acid 9 is a determinant of the import of PB2 into the mitochondrial matrix. Both N9 and T9 are compatible with mitochondrial matrix targeting of PB2, while the acidic amino acid D9 is not. Given the prevalence of these amino acid residues in influenza virus isolates, these results suggest that the PB2 of over 99% of all human seasonal pre-2009 H1N1, H2N2, and H3N2 influenza viruses is targeted to the mitochondrial matrix. On the other hand, the vast majority (over 95%) of avian influenza viruses encode nonmitochondrial D9-PB2 (18). Interestingly, PB2 is not the only influenza A virus protein that is differentially targeted to mitochondria. The localizations of PB1-F2, an auxiliary viral protein that has been shown to induce apoptosis in a cell type-dependent manner, promote inflammation, and regulate viral polymerase activity (39–41), also differ between influenza A virus strains. Most influenza A viruses encode full-length 87-to-90-aa PB1-F2 that associates with mitochondria due to the presence of an MTS in the C-terminal half of PB1-F2. However, all human pre-2009 H1N1 viruses isolated after 1947 encode a truncated, 57-aa-long, nonmitochondrial version, due to the presence of a stop codon, while pandemic 2009 H1N1 viruses and the resultant H1N1 human seasonal lineage encode a truncated 11-aa version (41, 42). These observations support the idea that mitochondria are actively targeted by influenza A viruses, mediated via viral proteins PB2 and PB1-F2, and that there might be differential evolutionary pressures for modulating mitochondrial function between different viral strains and between human and avian hosts.

We also showed that interaction of PB2 with MAVS is not a determinant of its mitochondrial association; PB2 of seasonal human influenza viruses shows the same pattern of mitochondrial localization in MAVS knockout cells as in control cells expressing MAVS. If PB2 is not recruited to the mitochondria through its binding to MAVS, questions arise regarding which other mechanisms are used to target PB2 to the mitochondria and how it is imported into the mitochondrial matrix. Our group proposed that the N-terminal α-helix of PB2 acts as an MTS that contains a number of hydrophobic and positively charged amino acid residues, hallmarks of amphipathic helices implicated in mitochondrial targeting (16). Indeed, mutations of two hydrophobic residues, L7 and L10, were found to inhibit PB2 mitochondrial localization, and the host-specific amino acid residue at position 9 is a determinant of mitochondrial localization, strongly suggesting a key role for the N terminus of PB2 in mitochondrial matrix targeting. Mitochondrial matrix proteins that are translated in the cytoplasm are targeted to the translocase of the outer membrane (TOM) complex, which acts as a general entry gate for protein import into the mitochondria by mediating protein transfer into the intermembrane space. After passage through the TOM channel, proteins destined for the mitochondrial matrix are imported by the translocase of the inner membrane TIM23 complex and released into the matrix (43). Recently, our group identified a range of chaperones and mitochondrial proteins, including Tom22 and Tim50, that copurify with PB2 from influenza virus-infected cells (44). Tom22 is part of the TOM complex, and it directly recognizes the hydrophilic face of the MTSs and facilitates the transport of the cargo protein through a channel formed by Tom40, another subunit of TOM, across the outer mitochondrial membrane. Tim50 is part of the TIM23 complex and acts as a primary receptor for proteins delivered by the TOM complex. It mediates protein transfer toward the translocation pore formed by Tim23 in the inner mitochondrial membrane. We therefore propose that PB2 is delivered to the mitochondria with the assistance of the Hsp70 and Hsp90 chaperones (44–47) and that its translocation into the mitochondrial matrix is then mediated by Tom22 and Tim50 of the mitochondrial import pathway. However, further studies are required to address the issue of exactly which mechanisms are operative in transport of PB2 into the mitochondrial matrix.

The finding that PB2 is imported into the mitochondrial matrix raises further issues about the functional implications of the mitochondrial association of PB2. As PB2 is imported into the mitochondrial matrix, mitochondrial PB2 is unlikely to interfere with MAVS function by directly binding to it as previously suggested (15). Although both the mitochondrial and nonmitochondrial forms of PB2 have been found to inhibit MAVS-mediated IFN-β expression (25), previous work from our group suggested that mitochondrial association of PB2 is nevertheless an important factor in determining its effect on IFN-β expression. In particular, a mitochondrial form of PB2 was able to inhibit IFN-β expression to a greater extent than a nonmitochondrial form (15). In support of this, a recombinant influenza virus encoding nonmitochondrial PB2 induced higher expression of IFN-β in cell culture and resulted in the attenuation of virulence in a mouse infection model (15). These observations suggest that mitochondrial association of PB2 is an important factor that could influence innate immune responses and pathogenicity.

How could PB2 localized to the mitochondrial matrix affect MAVS function and innate immune responses? PB2 could interfere with MAVS function by affecting the MMP, in a way similar to that proposed for influenza A virus protein PB1-F2, which is also targeted to the mitochondria and inhibits MAVS-mediated interferon expression by decreasing MMP (48). PB1-F2, imported into the mitochondrial intermembrane space, causes depolarization of MMP either by directly forming membrane pores (49) or by interacting with ANT3/VDAC (50). Maintenance of intact MMP is important for MAVS-mediated interferon production (51), and PB1-F2 has been reported to enhance the interferon-antagonist function of PB2 (48). Indeed, previous work by our group implicated PB2 in affecting MMP (16). Furthermore, our group identified TUFM as an interactor of PB2 (44). TUFM, a mitochondrial protein localized to the mitochondrial matrix, has been reported to regulate virus induced interferon expression in complex with NLRX1 (52). Thus, it is possible that mitochondrial matrix PB2 interferes with MAVS function indirectly through affecting MMP and/or through its interaction with TUFM. Interestingly, we observed a reduction in mitochondrial size and an increase in mitochondrial numbers in cells expressing mitochondrial PB2 compared with cells expressing the nonmitochondrial form. This could have been due to the mitochondrial PB2 disrupting the mitochondrial fusion/fission equilibrium through affecting MMP. Reduction of MMP has been linked to the inhibition of mitochondrial fusion and subsequent mitochondrial fragmentation (38). Although we see clear effects of mitochondrial PB2 on the size of mitochondria, the exact mechanisms by which PB2 affects the MMP and the fusion/fission equilibrium as well as any effects of PB2 on the major function of mitochondria, oxidative phosphorylation, remain unclear and await further investigations.

We also demonstrated here that an influenza A virus encoding a mitochondrial matrix localizing PB2 replicates to higher titers in MEFs than an isogenic virus encoding a non-matrix-localizing PB2. This is in agreement with previous data showing similar differences between the two viruses in a mouse infection model (15). We show here, however, that this difference is not solely due to the role of PB2 in inhibiting the expression of interferon, as it was also observed in MAVS knockout MEF cells, which are deficient in interferon expression mediated by RIG-I and MDA-5. This indicates that mitochondrial matrix-localized PB2 might have a second, undocumented effect on mitochondrial function which is important for influenza A virus replication. It is, however, also possible that this effect could be due to differences between the functions of N9-PB2 and D9-PB2 containing influenza virus polymerase (15). The functional significance of the mitochondrial matrix localization of PB2 requires further investigation using different viral strains of different subtypes in both cell culture and animal models.

Recently, two novel PB2-related proteins, expressed in influenza A virus-infected cells, have been identified. PB1-S1 is encoded by a spliced version of the PB2 mRNA, resulting in a C-terminally truncated form of PB2 that contains the N-terminal 495 amino acids of PB2 fused to a 13-amino-acid sequence expressed from an alternative open reading frame (25). In agreement with the presence of the MTS residing at the N terminus of PB2, PB2-S1 has been reported to localize to mitochondria and inhibit IFN-β expression. PB2_Δ_, another PB2-related protein that is detectable only in cells infected with a particular strain of H5N1 avian influenza virus, is encoded by a defective PB2 segment with an internal deletion that results in a 71-amino-acid N-terminal fragment of PB2 fused to a 19-amino-acid sequence derived from an alternative open reading frame (29). Interestingly, in contrast to full-length PB2 and PB2-S1, this PB2 fragment, which also associates with mitochondria, was observed to induce IFN-β expression, causing a reduction of viral replication in cell culture. However, the virus caused enhanced disease severity in the mouse infection model, possibly due to the amplification of early innate immune responses. These study results further highlight the importance of PB2 and its derivatives in affecting innate immune responses to influenza virus infection.

In summary, we show here that over 99% of human seasonal influenza A virus pre-2009 H1N1, H2N2, and H3N2 strains encode a PB2 protein that is targeted to the mitochondrial matrix. Although PB2 interacts with MAVS, its targeting into the mitochondrial matrix is MAVS independent. The N-terminal MTS is responsible for the mitochondrial matrix targeting of PB2, and we suggest that PB2 is imported by using the classical mitochondrial import pathway. This report highlights potential mitochondrial matrix-associated functions of PB2 which could be important in the replication and pathogenesis of influenza A viruses.
